# Why patients decline participation in an intervention to reduce re-hospitalization through patient activation: whom are we missing?

**DOI:** 10.1186/s13063-019-3187-9

**Published:** 2019-01-25

**Authors:** Maria Flink, Carina Brandberg, Mirjam Ekstedt

**Affiliations:** 1 Department of Learning, Informatics, Management and Ethics, Karolinska Institutet, Stockholm, Sweden; 2 Department of Social work, Karolinska University Hospital, Stockholm, Sweden; 3 Department of Health and Caring Sciences, Linnaeus University, Kalmar, Sweden

**Keywords:** Self-care, Re-hospitalization, Randomized controlled trial, COPD, Heart failure, Patient activation intervention

## Abstract

**Background:**

Despite worldwide interest in reducing re-hospitalization, there is limited knowledge regarding characteristics of patients who chose to decline participation in such efforts and why. The aim is to explore reasons to decline participation in an intervention using motivational interviewing to reduce re-hospitalization through patient activation for persons with chronic obstructive pulmonary disease or heart failure.

**Methods:**

This study uses data from 385 patients who were asked about participating in a randomized controlled trial; of these, 232 declined participation. Data on age, gender, and diagnosis were collected for those who agreed to participate and those who declined. Reasons to decline participation were collected for those who were asked to participate but refused. The stated reasons to decline were analyzed using content analysis, and the categories identified were used for the statistical analysis.

**Results:**

The main reasons for declining participation were having sufficient support (17.5%), no need for support (16%), being too ill (14.6%), and lack of time for illness-related activities (14.2%). A statistically significant negative association between age and willingness to participate was found (odds ratio = − 0.03, 95% confidence interval 0.95–0.99).

**Conclusions:**

Those who agreed to participate were younger than non-participants, and non-participants either lacked time for illness-related activities or did not have the energy needed to become involved in the intervention.

**Trial registration:**

ClinicalTrials.gov, NCT02823795. Registered on 1 July 2016.

## Background

In recent years, an increasing number of interventions have aimed to reduce re-hospitalization rates for patients with various health conditions [1, 2]. The main concerns have been the increasing costs due to the readmission rates. For example, a quarter of patients with heart failure are usually readmitted to hospital 30–90 days after hospitalization in the USA [3], and although the figures are lower in many European countries, 21% were readmitted within 90 days after discharge in a Swedish study of geriatric patients [4]. The huge efforts to address the large proportion of readmissions have raised a question of whether reduced re-hospitalization truly is a measure of quality [5]. The USA statutorily mandated federal Hospital Readmissions Reduction Program, promoted by penalties from 2013, showed limited effects on the 30 days readmission rates but unintended increased mortality 30 days, 90 days, and 1 year following hospitalization [6]. Still, many interventions aim to improve the transition to home through either single or multiple efforts, e.g., case management, discharge planning, or patient activation in self-care [1, 2], of which support for patient activation in self-management was found to be among the most efficient [2]. Patient activation is described as a person’s “knowledge, skills and confidence to manage one’s health and health care” [7]. Patients with high levels of activation have the most effective self-management skills, and evidence also illustrates that those with high knowledge of both their disease and its management have lower re-hospitalization rates [8–11].

A large proportion of the care transition interventions recruit patients at the interface between hospital care and the patients’ return to home. The patients are thus asked about their willingness to participate in the interventions at a time when they are particularly vulnerable. Multiple qualitative studies have shown that both patients and professionals consider the discharge encounters as a difficult time to give information, as patients are often overwhelmed by information and stressed about going home [12, 13]. In line with this, several studies report large numbers of patients who refuse to participate in the interventions. For example, Naylor et al. [14] reported 43% refusing participation and an age difference between enrollees and refusals. The same authors acknowledged in another RCT that the main reason for patient or family member refusal was due to an already-established relationship with a home health agency [15]. Coleman et al. [16] report 24% and Davis et al. [17] report 37% of patients refusing participation, but provide no further information about refuser characteristics. However, other similar studies have not reported information on the number of people who refused participation [18, 19] or reported only very small numbers of refusals [20].

Despite the worldwide interest in interventions that could help reduce re-hospitalization rates by increased patient activation [2], there is limited knowledge on who declines participation and why. Such knowledge could help target future interventions more carefully to the population, for example, in terms of when the patients are approached and how. It is also of importance in the implementation of interventions in real-life settings, as the external validity may be affected when a large proportion of patients decline participation [21]. The aim is to explore reasons to decline participation in an intervention using motivational interviewing (MI) to reduce re-hospitalization through patient activation for persons with chronic obstructive pulmonary disease (COPD) or heart failure (HF).

## Methods

This study uses data from participants who were asked about participating in an ongoing randomized controlled trial (RCT) in Sweden. The RCT aims to reduce re-hospitalization rates for patients with COPD or HF using MI to improve patient activation for self-care [22]. The intervention consisted of five sessions: one face-to-face (1 week post-discharge) and four telephone sessions during a time span of 4 weeks post-discharge. Patients allocated to the intervention group received MI sessions by a patient activation coach with the overarching goal that the patient becomes motivated to be active in post-discharge self-management. More details of the intervention can be found in the study protocol [22]. The present study uses data from the first 9 months of the RCT.

### Recruitment and consent process in the RCT

Patients with COPD or HF were approached during hospitalization for any cause at any of the six eligible internal medical wards at two hospitals (one university hospital and one general hospital). Eligible patients (diagnosed with COPD or HF, able to speak/understand Swedish, living at home, no diagnosis of dementia or mild cognitive impairment, no “do-not-resuscitate” statement in medical record) were identified by a nurse or physician at the wards. The recruitment was conducted by one of two researchers (MF and CB) as follows: The researcher approached each eligible patient in hospital and asked for permission to inform them about this study. If the timing was bad, for example, the patient was engaged in some activity or too tired, permission was asked to come back later the same, or another day. The information given, if permission was granted, consisted of reasons for conducting the study and that the goal was to strengthen the participants’ self-care ability at home, post-discharge. Patients were also informed that if they accepted participation, they would be randomized to either a control group or an intervention group and that those in the intervention group would interact with a coach. The patients in the control group would receive care as usual. Further, patients received information about the questionnaires and got the chance to look it over before consenting to participate. The researchers asked patients about their perceived ability to complete the questionnaire and possibilities of posting the questionnaire in the provided envelope. Participants with difficulties completing the questionnaire by themselves (e.g., due to poor eyesight, difficulty writing, or just a need to discuss the questions) were offered help in filling out their answers. Patients who agreed to participate were given the baseline survey and were thereafter randomized to intervention or control. The questionnaires at baseline consisted of the Morisky Medication Adherence Scale, Patient Activation Measure, EuroQol five dimensions (EQ-5D), Patient Health Questionnaire (PHQ-9), and the Need Satisfaction and Frustration Scale (NSFS). An average time to fill out the questionnaires was 30–40 min.

### Data collection

Patients who declined participation were asked their year of birth, why they declined participation, and if we could return to them later for a short telephone interview. The reason for declining participation was noted by the researchers verbatim (as much as possible), along with patient gender and diagnosis. The data hence consist of gender, age, and diagnosis for both patients who agreed to participate and those who refused participation, as well as reasons for declining participation for those who refused.

We contacted five randomly selected patients about 1–3 months after the initial recruitment initiative, after the patients had returned home, to ask if they were willing to participate in the telephone interview. All of them accepted participation. Short semi-structured telephone interviews were conducted as a quality assessment to explore if the patients had changed their reasons for refusal after returning home.

### Analysis

The stated reasons to decline participation were analyzed using qualitative content analysis [23]. All statements were transcribed into a file in Excel, and each statement was coded. When multiple reasons were stated, the reason that was emphasized in the statement was chosen to be coded. For example, for one patient it was stated “Has home healthcare and a wife. Consider that he gets all the support he needs. Also has difficulties to talk.” In this case, the code “already has support” was given. The codes were sorted into sub-categories and categories based on similarities (Table 1). The overall analysis was conducted by MF with the assistance of ME. Disagreements between MF and ME were solved through discussion. CB read through and commented on the analysis.

**Table 1:** Categories and sub-categories

Category	Sub-category	Examples of stated reasons
No need for the intervention	Having enough support	Has home healthcare that are responsible for his medications and has social services. Thinks that this is enough support Thinks that she has enough support from her children and grandchildren. Two of the children are physicians who help her. Says that she is fortunate in this sense
	Having no need for support	Doing just fine at home Has been to therapy a couple of years ago and then got all the tools he needed to change things in his life. Has no need for any support
	The study concept not suiting the patient	Talking doesn’t help at all I have participated in similar things before and it didn’t help at all. Not interested in talking about my situation
	Not believing they have the diagnosed disease	Doesn’t think that she has COPD Doesn’t feel ill at all and questions that she really has COPD; the study doesn’t seem relevant to her based on this
Can’t manage more tasks	Being too sick	Doesn’t have the energy. Too ill right now. Maybe another time No energy and a lot of trouble with breathing. Gets really tired and breathless by talking on the phone
	Not having time for more illness-related activities	Has too many activities scheduled with her COPD; does not want to have more on her schedule Has too much with her dietician, physician, nurse, and so on. Feels stressed over all this; does not want any more stress
	Taking care of sick relatives	Has too much to do with her husband who has dementia Her husband with Alzheimer’s lives at home with her. He doesn’t want to move and doesn’t want any help from others. She doesn’t want to add any more tasks
	Not trusting their own ability to participate	Says that he doesn’t understand everything, doesn’t have the energy to participate, and has trouble hearing Has had a stroke which has affected his cognitive abilities; doesn’t want to participate based on this
	Being in a crisis	Has just got her diagnosis and is upset over this and the situation of her husband, whom she has cared for until now A close relative just passed away
Other	Refusing because this was a research study	Has already participated in two research projects; doesn’t have the energy to participate in one more Doesn’t like questionnaires
	Practical reasons	Will be out of town for a few months Doesn’t live in Sweden
	Unknown reasons	–

The sub-categories and categories were used for statistical analysis. Pearson’s chi squared test and descriptive statistics (percentage, mean) were used to calculate differences between groups. Logistic regression was used to predict a possible relationship between patient characteristics and the dependent variable: acceptance of participation. IBM SPSS Statistics 24 was used for the statistical analysis.

## Results

**Fig. 1:**
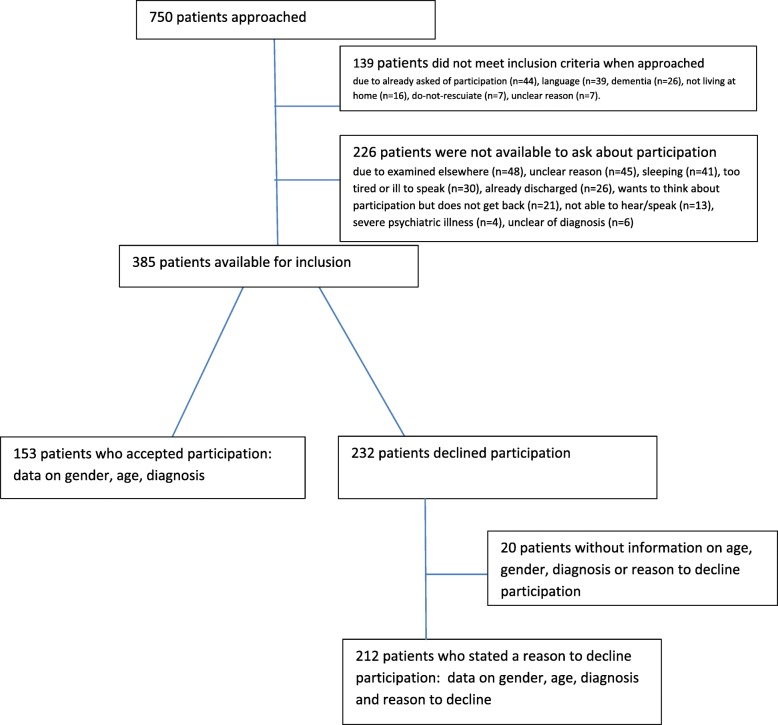
Flow chart

Over the course of 9 months, from August 2016 to May 2017, 750 patients with COPD or HF were screened for inclusion after hospitalization for any cause (Fig. 1). Of these, 365 patients (48.7%) were not asked about willingness to participate as they either did not meet the inclusion criteria when approached (*n* = 139) or were not available for recruitment (*n* = 226). The main reasons for not meeting inclusion criteria when approached were as follows: did not speak Swedish or understand the verbal study information (*n* = 39), had already declined willingness to participate during a previous hospitalization period (*n* = 44), or showed signs of dementia/cognitive impairment which were confirmed by other healthcare professionals (*n* = 26). The main reasons for not being available for recruitment were sleeping (*n* = 41), examined elsewhere in the hospital (*n* = 48), or too tired or ill to speak (*n* = 31). The remaining 385 patients were asked about their willingness to participate in the RCT. Of these, 153 agreed to participate and 232 refused participation. In 20 cases, no information at all was collected on patients who refused participation because the researchers missed taking notes. Of the 212 patients declining participation for whom data were obtained, 124 had HF, 74 had COPD, and 6 had both HF and COPD. For eight patients, data on diagnosis were not collected (Table 2).

**Table 2:** Reasons to decline participation according to gender, age, and diagnosis

Category	Sub-category	All, *n* (%)	Women, *n* (%)	Age, mean (range)	HF, *n* (%)	COPD, *n* (%)	HF and COPD, *n* (%)
No need for the intervention	No need for support	34 (16)	18 (16.4)	77.6 (51−93)	26 (21.0)	8 (10.8)	–
	Having sufficient support	37 (17.5)	17 (15.5)	77.4 (56−91)	24 (19.4)	10 (13.5)	1 (16.7)
	The study concept not suiting the patient	14 (6.6)	8 (7.3)	73.7 (33−85)	5 (4.0)	7 (9.5)	2 (33.3)
	Not believing they have the diagnosed disease	5 (2.4)	5 (4.5)	72 (60−77)	–	5 (6.8)	–
Cannot manage more tasks	Being too ill	31 (14.6)	19 (17.3)	80.3 (68−95)	17 (13.7)	9 (12.2)	2 (33.3)
	Being in a crisis	7 (3.3)	5 (4.5)	79.3 (68−88)	4 (3.2)	3 (4.1)	–
	Not trusting their own ability to participate	15 (7.1)	7 (6.4)	79.4 (60−97)	8 (6.5)	7 (9.5)	–
	Not having time for more illness-related activities	30 (14.2)	12 (10.9)	77.6 (57−93)	22 (17.7)	7 (9.5)	1 (16.7)
	Taking care of sick relatives	5 (2.4)	4 (3.4)	83.6 (70−93)	2 (1.6)	3 (4.1)	–
Other	Refusing because this was a research study	24 (11.3)	11 (10.0)	78.9 (67−98)	10 (8.1)	12 (16.2)	–
	Practical reasons	5 (2.4)	1 (0.9)	72.8 (62−81)	3 (2.4)	2 (2.7)	–
	Unknown	5 (2.4)	3 (2.7)	81.3 (72−93)	3 (2.4)	1 (1.4)	–
	Total	212	110	–	124	74	6

A statistically significant association was found between age and acceptance of participation; the acceptance decreased with age (odds ratio, OR = − 0.03; 95% confidence interval, CI 0.95–0.99) adjusted for gender. The assumption of linearity to the logit was fulfilled. The mean age for those accepting participation was 75.3 years (range 40 – 98; standard deviation, SD 10.1), and the mean age for those declining participation was 78 years (range 33–98, SD 9.2). We did not observe statistically significant differences in gender between those who accepted participation and those who declined (*p* = 0.940) or between patients with different diagnoses (*p* = 0.065). A larger proportion of patients with HF declined than accepted participation (60.5% vs. 39.5%).

The overall reasons to decline participation were “No need for the intervention” (*n* = 90, 42.5%) and “Can’t manage more tasks” (*n* = 88, 41.5%); see the details in Table 2. Those who stated that they could not manage more tasks were older than those who did not perceive that they had a need for the intervention.

The short semi-structured interviews found the same reasons for declining participation at hospital as after the return home. When asked open questions on why they chose not to participate, the patients stated similar reasons as at the hospital.

## Discussion

This short paper addresses a concern regarding who declines participation and why, in an intervention recruiting patients at hospital discharge to reduce re-hospitalization through increased activation. Those who accepted participation had a lower mean age than those who declined participation. The main reason for declining participation was having sufficient support. However, 41.5% of the population who declined participation perceived that they could not manage more healthcare-related tasks due to their own or their relatives’ health situation.

Almost one fourth of the population (22.9%) asked about willingness to participate felt that they could not manage any more illness-related activities at the time when they were offered the intervention. This may suggest that the timing of interventions aiming to increase patient activation for and participation in self-care activities might be an important factor to consider, as the patients recruited for interventions may be too ill to manage participation. This can also be related to the hospitalization period. As the length of hospital stays is short in Sweden (5.7 days on average) as well as in other comparable countries [24], and the number of hospital beds is decreasing [25], it is possible that people are discharged before they feel well enough to consider participation in clinical trials. The timing may also be important to consider in relation to the patient’s age, as the patients who agreed to participate had a lower mean age than those who declined. This could mean that interventions aiming to increase patients’ activation in self-care should target patients early in their care trajectory when they potentially have more energy and fewer health-related activities. .

Our study does not provide any information as to whether non-participants would have made another decision if they had been given the opportunity to participate after a few weeks at home or earlier in their care trajectory. Studies suggest that to increase days at home for frail elderly, these patients would benefit from a thorough discharge planning and more intensive follow-up at home [14]. Thus, future trials investigating healthcare usage rates might change focus to avoiding hospitalization instead of decreasing re-hospitalization, for example, by approaching patients in primary healthcare or other out-patient settings. The large number of patients reporting that they did not need the intervention also calls for designing interventions in close collaboration with patients to tailor them to “what matters for the patient” [26].

One limitation of this study is the telephone interviews. The interviews were not audio-recorded, and only handwritten notes were taken on the reasons why the patients declined participation. The telephone interviews were conducted to explore if the patients changed their mind regarding reason to decline once they had returned home. Only five interviews were conducted, as all five expressed similar reasons as at the hospital. However, based on this, we do not know if other patients might have added more in-depth understanding of reasons to decline participation if more and longer interviews had been conducted. Another limitation is that many patients were not available to be asked about participation, due to practical reasons with limited available dates for data collection by the researchers. There is a risk that those unavailable have a different proportion of participants and non-participants than in the studied sample.

## Conclusions

This study casts light on who chooses to accept or decline participation in interventions at hospital discharge. Participants were younger than non-participants, and non-participants either lacked time for more illness-related activities or the energy needed to become involved. Future studies aiming to decrease healthcare usage might consider recruiting patients in primary healthcare, hence changing focus to avoiding hospitalizations instead of re-hospitalizations.

## Abbreviations

COPD: Chronic obstructive pulmonary disease
HF: Heart failure
RCT: Randomized controlled trial
